# Valorization of jackfruit waste into value added products and their potential applications

**DOI:** 10.3389/fnut.2022.1061098

**Published:** 2022-11-29

**Authors:** Neelam Pathak, Sangram Singh, Pankaj Singh, Pradeep Kumar Singh, Ranjan Singh, Saroj Bala, Banjagere Veerabhadrappa Thirumalesh, Rajeeva Gaur, Manikant Tripathi

**Affiliations:** ^1^Department of Biochemistry, Dr. Rammanohar Lohia Avadh University, Ayodhya, Uttar Pradesh, India; ^2^Biotechnology Program, Dr. Rammanohar Lohia Avadh University, Ayodhya, Uttar Pradesh, India; ^3^Department of Microbiology, Dr. Rammanohar Lohia Avadh University, Ayodhya, Uttar Pradesh, India; ^4^Department of Microbiology, Punjab Agricultural University, Ludhiana, Punjab, India; ^5^Microbial Processes and Technology Division, Council of Scientific and Industrial Research (CSIR)-National Institute for Interdisciplinary Science and Technology, Thiruvananthapuram, Kerala, India; ^6^Academy of Scientific and Innovative Research (AcSIR), Ghaziabad, Uttar Pradesh, India

**Keywords:** jackfruit waste, biofuels, microbial fermentation, valorization, environmental sustainability

## Abstract

Jackfruit is a potential natural resource for many valuable biomaterials. The wastes from jackfruit are rich in carbohydrate, proteins, fats and phytochemicals. These wastes can be used as feedstock for the development of various bioproducts. The pretreatment strategies like biological, physical and chemical methods are being used for effective valorization of fruit wastes into value added products, like bioethanol, biogas, bioplastics, feeds, functional food additives, and other useful compounds. Bioenergy production from such renewable resources is an eco-friendly and cost-effective alternative option of fuels, unlike fossil fuels. The efficient bioconversion of fruit waste into useful biomaterials is facilitated by microbial fermentation process. Also, jackfruit peel is applied in the pollution abatement by remediation of dyes color from contaminated aquatic environment. Such technology can be used to develop a green economic model for waste utilization. This review addressed the utilization feasibility of jackfruit waste to produce value added products in order to reduce wastes and protect environment in a sustainable way.

## Graphical Abstract

Valorization of jackfruit waste into value added products and their potential applications. 
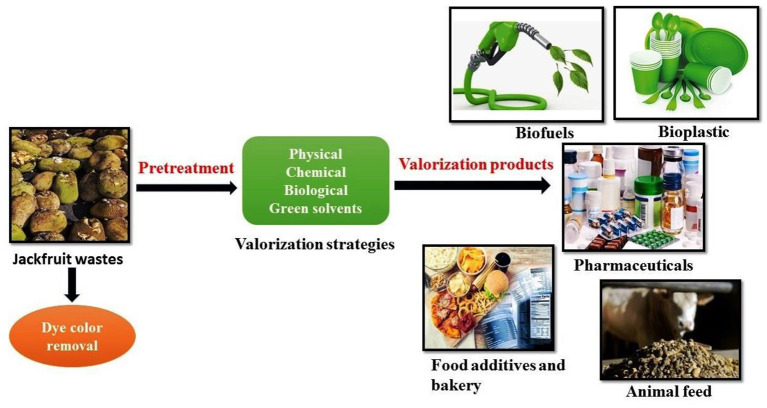

## Introduction

**Figure 1:**
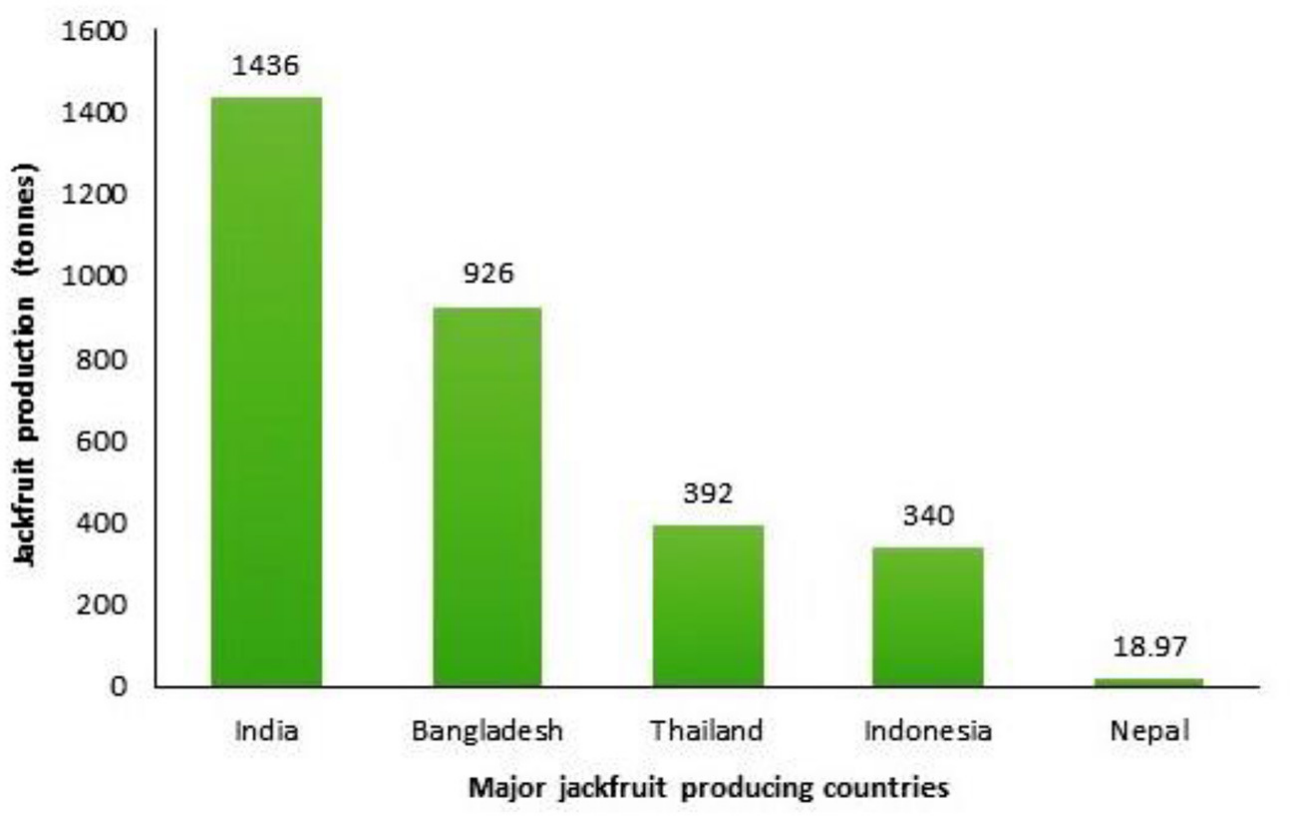
Major jackfruit producing nations. Sources: Haq (5) and APAARI (6).

The jackfruit (*Artocarpus heterophyllus*), belongs to Moraceae family and is a massive, edible tropical fruit that grows on trees (1). Vitamins, flavonoids, protein, minerals, antioxidants, and digestible starch are some of the nutrients found in abundance in the fruit, which makes it as a healthy food option (2, 3). Jackfruit grows especially in wet, humid coastal regions with plenty of annual rainfall (4). With a total output of 1.4 million tons, India is the top producer of jackfruit following Bangladesh, which recognizes jackfruit as its national fruit, ranks second, with a total output of 926 tons (Figure 1). Thailand, Indonesia, and Nepal are also significant jackfruit producers (5, 6). Jackfruit spoils quickly if it isn't eaten or preserved within a few days of being picked (7). It is an underutilized crop (8), but now its demand has increased because of its nutritional benefits. It was reported that the fit part of jackfruit for human consumption is ~25–35%, whereas the waste contributes to be 75–65% (9). The non-consumable portions of this fruit contain a central core, rind, and perianth (10).

Fruit waste is one of the major global environmental concerns. The outermost covering of jackfruit is termed as the peel that contribute ~50% of the ripen fruit (11). However, the golden-yellow pulp is edible portion and is arranged in fleshy bulbs, each bulb contains a single seed (12), these are sources of minerals, vitamins and bioactive compounds. But, the massive amount of discarded jackfruit is a serious ecological problem in jackfruit-producing nations (13). According to estimates, a single jackfruit tree can produce fruit averaging between 5 and 7 kilograms in weight. Jackfruit waste has been investigated for its potential to be converted into a wide variety of bioproducts, including biofuels, animal feed, bioactive components, bakery industry, and bioplastics. Fruit wastes are valorized into value-added products using physical, chemical, biological, and environmentally friendly pretreatment methods (14, 15).

The problems due to fruit wastes can be solved by turning them into valuable goods, such as increasing the health benefits while reducing the amount of organic matter in the environment (16). Therefore, one of the most important ways to utilize jackfruit waste components is to produce several valuable bioproducts through valorization process. However, the pretreatment and extraction processes are important steps for successful and effective valorization of such wastes. The aim of this review is to address valorization technologies to develop a variety of useful products from jackfruit waste and their uses in different sectors.

## Biochemical composition of jackfruit and its waste

Jackfruit is broadly accepted by researchers, food industries, and consumers due to the presence of important phyto-nutrients and phyto-chemicals (9, 17). According to Akter and Haque (8), about 70–80% are non-edible and ~60% of which correspond to the outer rind, perianth, and central core of jackfruit and are considered as waste. Biochemical composition of jackfruit and its waste showed that it can provide health benefits, and being an ecofriendly raw resource for production of many valuable bioproducts. The peel of jackfruit contains cellulose, protein, starch, and pectin, respectively (18). Kumar et al. (19) reported that dried seeds of jackfruit contain carbohydrate (76.1%), protein (17.8%) and lipid (2.1%). Furthermore, Sumathy et al. (20) reported many phytonutrients, isoflavones, lignin, saponins and many other important nutrients in jackfruit seeds. Similarly, Fernandes et al. (21) reported that jackfruit seeds are the excellent sources of vitamins like thiamine, and riboflavin. Jackfruit peels, seeds, and latex are among of the solid wastes produced during its processing, which contribute to environmental damage (22). These fruit wastes can be used as an environmentally friendly source for many sustainable products due to the chemical composition of waste biomass which may be used as feedstock for bioproducts development. Researchers reported that jackfruit peel can be used as an alternative source of commercial pectin, biofuels and other bioproducts in a sustainable way (22).

## Valorization technologies of fruit waste for value-added product formation

**Figure 2:**
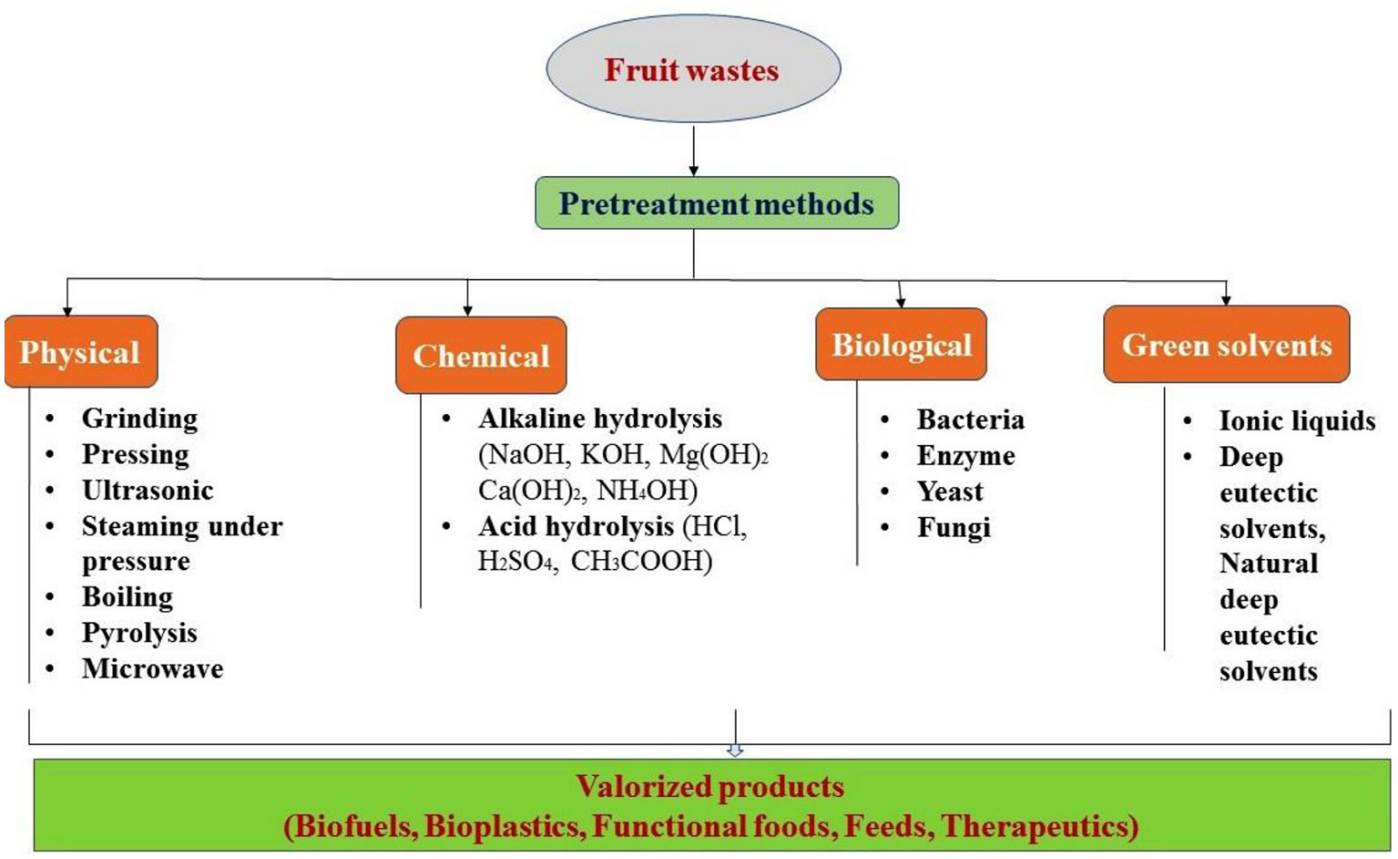
Pretreatment strategies for effective valorization of fruit wastes into value added products.

Lignocelluloses are one of the major renewable and cost-effective resources for bioproducts development. Valorization is an environmentally friendly and sustainable approach in which the waste biomass either recycled or converted into valuable products. In recent past, researchers reported different valorization routes for development of value-added chemicals (23). The pretreatment strategies employed for effective valorization of waste biomass into useful products are discussed as following (Figure 2).

### Physical

The term “physical pretreatment” refers to the utilization of mechanical methods such as size reduction, pressure, pyrolysis, boiling, microwave, and ultrasonication (24). Pretreatment is a crucial step in the conversion process, as it modifies the jackfruit wastes into simpler forms so that more of the fermentable sugars in the carbohydrate polymers are accessible to the enzymes that catalyze the reaction. Reports indicated that ethanol extracted from jackfruit seeds under low pressure. Resendiz-Vazquez et al. (25) looked into how high-intensity ultrasound affected the composition and functionality of an isolated protein after extracted from jackfruit seeds. Jackfruit can be processed using a wide variety of physical methods to develop valuable bioproducts (26). These methods include irradiation, microwave processing, supercritical fluid extraction, high-pressure processing, and others advanced physical pretreatment technologies (24, 26).

### Chemical

Jackfruit biomass is chemically pretreated by first mixing it at temperatures ranging from 130°C to 210°C, then adding concentrated or diluted acids and bases. The hydrolysis of the sugars can take from a few minutes to a few hours, depending on the pretreatment conditions (27). The effects on methane production, energy potential, and environmental benefits were studied by Umeghalu (28), using jackfruit peel. The effects on methane generation through pretreatments of jackfruit with 5% alkaline hydrogen peroxide (AHP), which increased methane yield and biodegradability by 69.8% compared to untreated, were investigated, and there is a potential for annual energy production from jackfruit peel treated with 5% AHP (29). In a separate investigation, Choy et al. (30) used alkaline extraction methods for successful isolation of starch from locally grown jackfruit seeds, achieving a yield of ~18%. These extracted chemicals can be further served as a feedstock for development of various useful bioproducts.

### Biological

Conventional chemical and physical pretreatment techniques call for high investment in reagents, machinery, and energy. However, biological pretreatment of cellulosic and lignocellulosic materials before enzymatic saccharification, on the other hand, employs the use of living microorganisms (bacteria and fungi) and has less effect on the environment and energy consumption (31). Many microorganisms, both cellulolytic and hemicellulolytic, which exist in nature can be used. Yeast (*Saccharomyces cerevisiae*) is essential for the production of ethanol from jackfruit. Ethanol contains ~35% oxygen, so when burned, it produces fewer nitrogen oxides and particulate matter than gasoline does (32). Bioethanol may be produced through enzymatic hydrolysis, and microbial fermentation of cellulosic biomass which is a cost-effective and environmentally friendly approach. Yuvarani and Dhas (32) used jackfruit peel as a feedstock for bioethanol production due to its high carbohydrate percentage. Researchers in another study investigated the possibilities of saccharifying jackfruit rind using a recombinant enzyme endoglucanase from *Bacillus subtilis* MU S1 at 50°C, pH 5.0, and 15 mg/ml of the substrate (33). These findings clearly indicate that recombinant enzyme of *B. subtilis* can easily be used for saccahrificaion of lignocellulosic agro-wastes, a cost-effective technique for sugar production (33). In another study, Joyline and Aruna (34) investigated the capacity of *Bacillus megaterium* strain JHA, which was isolated from oil-contaminated soil, and tested in the lab on glucose for polyhydroxyalkanoates (PHA) accumulation. Agro-industrial wastes, including jackfruit seed and other organic and inorganic compounds, were also found suitable for PHA accumulation (34). The *B. megaterium* strain was able to complete PHA biosynthesis by making use of all available substrates. Additional characterization revealed that the bioplastic layer formed was a PHA. There are several other viable applications of PHA in medical science, cosmetics and pharmaceutical products. Further research is required to develop a process parameter for more extracellular production of PHA on cheaper carbon and nitrogen sources in order to achieve an economically viable commercial process for producing biodegradable plastics along with other biopolymers which make it more effective and durable. Biological pretreatment is a promising and environmentally friendly sustainable method for converting lignocellulosic biomass into a more manageable form (31, 35). However, it is important to optimize the process variables that affect pretreatment to improve the enzymatic conversion of the waste biomass into simple and usable forms for bioproducts production.

### Green methods

Green chemistry is a relatively new concept that has recently been introduced into traditional pretreatment methods. In this pretreatment approach, numerous solvents, such as deep eutectic solvents (DES) can be used as environmentally friendly alternatives to traditional techniques (35). In a study, Azman et al. (36) investigated the effect of deep eutectic solvent to characteristics of pectin-based bioplastic from fruit based feedstock. In addition, the effective extraction agent presents a variety of opportunities that support the implementation of environmentally friendly technologies as an essential shift in the industry. Roy et al. (35) reported that the effectiveness of the DES-based pretreatment may also be greatly increased by using microwave and ultrasound. Further research is necessary for technological development in pretreatment process that make use of environmentally friendly solvents for effective valorization of waste biomass at large scale level.

## Applications of jackfruit's bioproducts

**Figure 3:**
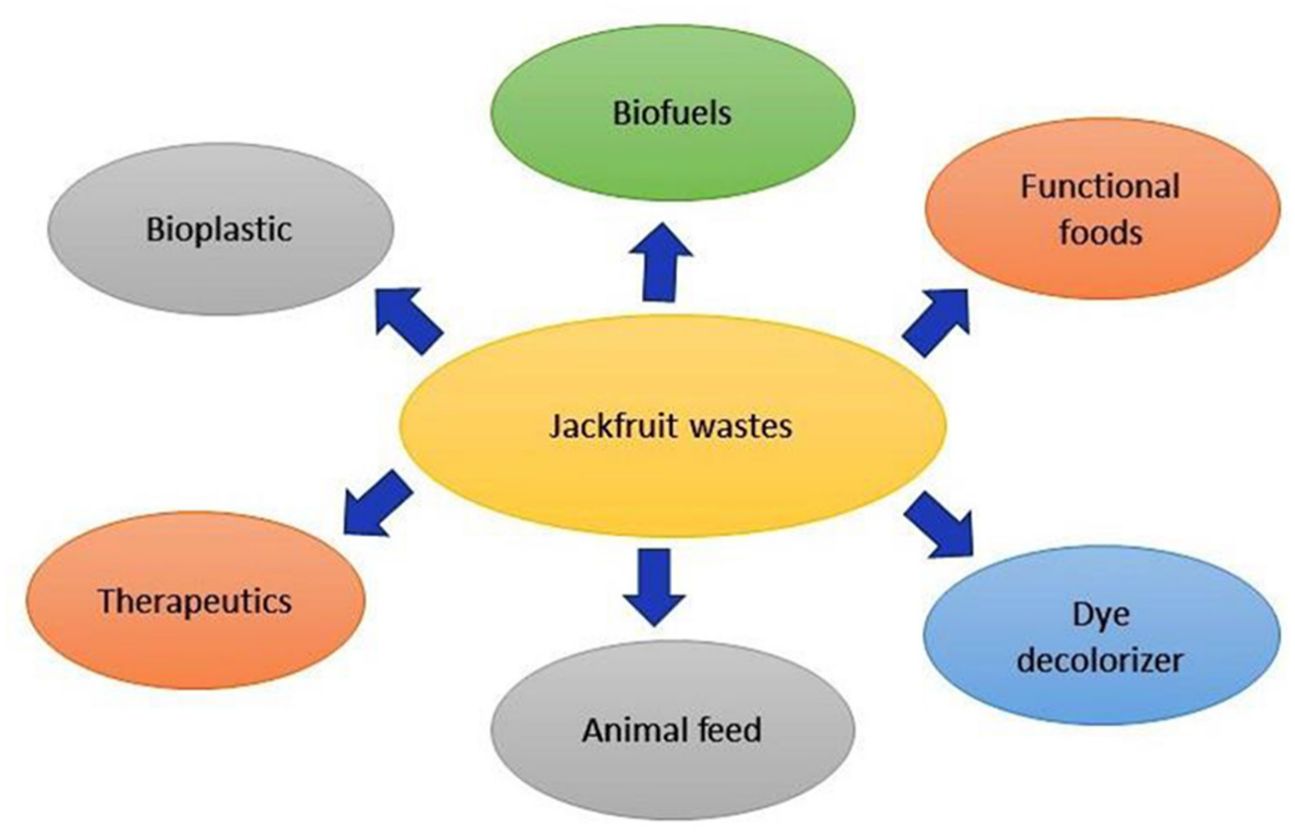
Different possible applications of jackfruit wastes.

Edible by-products include seeds and perianth, while non-edible wastes include peel and central axis from both fresh jackfruit consumption and its processing. Since the bio-based concept has a promising approach for improved efficiency, cost-effectiveness, better yield and environmental management, it is currently the primary focus of study. Some of the valuable bioproducts that can be made from jackfruit wastes are biofuels, functional foods, animal feed, biomaterials, bakery goods, drugs, and food additives (Figure 3). Biotransformation process has the potential to turn fruit wastes into valuable products.

### Bio-oil/fuels

Jackfruit peel waste is used to produce bio-oil, which was studied by Soetardji et al. (37) via a pyrolysis process in a fixed-bed reactor. A high number of volatile compounds were found in the peel after pyrolysis at high temperatures (400–700°C), suggesting that the biomass is a good resource for bio-oil production. Bio-oil that contains only trace amounts of sulfur (0.03%) and nitrogen (0.61%) is likely safe for the environment. At 550 °C, when the organic content was 85.2% and the water content was 14.8%, the biofuel quality was found to be optimal. With the help of a low-temperature pyrolysis method, discarded jackfruit peels can be converted into bio-oil. Using inexpensive adsorbents, Widjaja et al. (38) investigated the removal of water from bio-oil *via* the adsorption technique. Adsorption-based purification offers a low-cost, high-selectivity, and easy-to-implement alternative to other techniques. It has been found that the jackfruit peel oil obtained through the transesterification process is a viable option. The water content in bio-oil could be reduced after being purified with silica gel and zeolite, respectively (38). In another study, Yuvarani and Dhas (32) used *S. cerevisiae* for fermentation to produce bioethanol from cellulosic jackfruit peel biomass. Jackfruit peel composition, temperature, fermentation time, and nutrients availability all parameters are being studied to determine what would produce the best results, and for the ideal conditions for product development. These parameters might affect biofuel production from jackfruit peel. In another study, Ranasinghe and Marapana (39) reported biogas production through microbial fermentation process from jackfruit that contains carbohydrate as a feedstock. Biogas production involves anaerobic digestion of substrate which occurs via hydrolysis, acidogenesis, acetogenesis and methanogenesis (40). In an investigation, researchers used a feedstock material consisting of cow dung and jack fruit waste for biogas production, and found parameters like pH, temperature, carbon and nitrogen ratio might influence the anaerobic digestion process carried out by microorganisms (41).

### Animal feed

The jackfruit peel is highly recommended as a valuable raw material for animal feed due to its high levels of carbohydrate, protein, and fiber (8). The jackfruit waste supplementation improves feed intake and digestibility in ruminant. Ajay (42) researched jackfruit waste as a nutrient-enriched animal feed. Both crude protein and fiber were found to be highest in the jackfruit waste feed that was fermented using a combination of yeast and lactic acid bacteria supplemented with 2% ammonium sulfate. The dried powdered feed made from jackfruit waste contained moisture, carbohydrates, protein, crude fiber, crude fat, and ash. According to Kusmartono (43), jackfruit waste, which includes the peel and axis, has great potential as a ruminant feed.

### Bioplastic

The use of plastics developed from carbohydrate rich biomass can be an ecofriendly option compared to synthetic plastic. In a study, Kahar et al. (44) reported three different blend systems and found that plastic from jackfruit seed starch have thermal stability as well as the tensile properties. In another study, Lubis et al. (45) investigated bioplastics made of jackfruit seed starch plasticized with glycerol. Jackfruit seed starch plasticized with common plasticizers was also characterized by other researchers (46). Early tests showed that water, glycerol, sodium bicarbonate, and citric acid were among the plasticizers that worked well enough. After making four different types of bioplastics with different combinations of these plasticizers, the properties of these synthesized bioplastics were analyzed. Bioplastics made through biological processes using renewable biomass showed great promise as a material of the future due to their sustainability and high potential (46).

### Jackfruit waste as adsorbent

Recent remediation approaches through biological means is an effective way to remove environmental pollutants (47). Many researchers reported jackfruit peel for remediation of dyes color from aquatic environment (48, 49). Hameed (48) investigated peel of jackfruit for methylene blue dye removal with adsorption efficiency of 285.713 mg/g. The findings showed that an inexpensive adsorbent material could be made from jackfruit waste biomass. In another study, Jayarajan et al. (49) reported jackfruit peel as an adsorbent for remediating rhodamine from aquatic environment. This makes it a potential candidate that can remove dyes color in environmentally friendly way. According to Ahmed and Nasar (50), peels of jackfruit is a low-cost, promising adsorbent for removing methylene blue from aqueous solutions. The textile industry must use an appropriate treatment method for the removal of hazardous dyes from their discharged water. Even though the physico-chemical techniques are commonly used wastewater treatments, they are not very effective for removing some dyes (51). In another study, Miah et al. (52) investigated decolorization of a textile dye novacron blue using jackfruit seed with maximum adsorption of 0.732 mg/g after 1 h contact time. They found increasing dye level adversely affecting the extent of dye color removal. So, finding a cost-effective adsorbent material like jackfruit peel, available readily, is essential for remediation of dye color from polluted sites.

### Bakery products

Several researchers reported the use of jackfruit seed for production of flour. To make jackfruit seed flour, Akter (53) used drying technique, and prepared flour by grinding the dried seeds, and stored it in hermetically sealed containers (53). In another study, Abraham and Jayamuthunagai (54) made pasta from mixtures of jackfruit seed flour and wheat flour. They found that using a composite flour enhanced the pasta's nutritional value. In another study, Khan et al. (55) evaluated of quality characteristics of a mixed cake prepared using wheat and jackfruit seed flour. In another study, Butool and Butool (56) reported preparation of bread and biscuits using jackfruit seed flour. They found that when bread and biscuit recipes containing 10% and 20% jackfruit seed flour, the color and texture were superior. Biscuits made with jackfruit seed flour had higher ash and crude fiber content but lower carbohydrate content (56). When Aziz (57) mixed wheat flour with jackfruit seed flour to make bread, he found that the bread's fiber content increased while its protein content decreased slightly. Whereas, Hasidah and Noor Aziah (58) did similar work, and they added up to 25% jackfruit seed flour to bread and found that it was well received by a sensory panel. After much deliberation, they decided that jackfruit seed flour was a suitable alternative to wheat flour in some applications. Hossain et al. (59) experimented with combining jackfruit seed flour and wheat flour in varying ratios to bake bread. According to their findings, the bread made with a jackfruit seed flour substitution was the most preferable option.

### Pharmaceuticals and beauty aids

The starch and protein found in jackfruit seeds could be used in pharmaceuticals. Jackfruit seed starch is a super disintegrant used in the production of fast-acting tablets in which the tablets dissolve or disintegrate in the mouth without the need for extra water (60). In a study, Jitendra et al. (61) reported that jackfruit latex may be useful for treating different dental problems because it contains a lot of resin. The anticarcinogenic, antimicrobial, antifungal, anti-inflammatory, wound-healing, and hypoglycemic effects of jack fruits, leaves, and bark have led to their widespread use in traditional medicine. When used to make bio-nanocomposite materials for bone healing, jackfruit peel pectin has been shown to have better antimicrobial properties (1). The bionanocomposite made with 0.10 percent jackfruit pectin is suitable for orthopedic and orthodontic applications due to its good physicochemical and biological properties. Bone grafts are another possible application for this bio-nanocomposite (62). In a study, Pathak et al. (63) reviewed the therapeutic use of biopolymeric materials for disease cure. To better understand the biochemical effects and the overall nutritional benefits, Mandhare et al. (64) investigated the activity of compositions containing jackfruit extract and isolated phytochemicals for health benefits.

## Future perspectives and challenges

The development of valuable bioproducts can be accomplished in environmentally friendly manner and focused on valorization strategies that make use of jackfruit waste. Pharmaceuticals, the bakery industry, the production of biofuels and biomaterials, and others that fall under the umbrella of “interdisciplinarity” need to collaborate for the process to be successful. Utilizing eco-friendly pretreatment and integrating valorization technologies should be the primary ways to meet the current demands for biofuels, medicines, and other bioproducts that are produced using sustainable natural resources to protect the environment. Among the more recent pretreatment technologies for degradation, depolymerization through physical or chemical means, as well as conversion by microorganisms, are the methods that hold great potential. In the context of jackfruit cultivation, the multifaceted nature of jackfruit has increased interest in a variety of cutting-edge biotechnological approaches, such as molecular markers, omics technologies, and functional genomics. Several cutting-edge biotechnological approaches have emerged as a viable alternative to traditional jackfruit farming as a means of creating genetically modified jackfruit variants with enhanced agricultural production. Jackfruit waste can be diminished as a result of modern practices. This interest has been sparked by the fact that jackfruit can be used in a wide variety of applications. It has been demonstrated that jackfruits are capable of performing a diverse array of functions. Jackfruit wastes contain huge amount of carbohydrates of various nature along with protein and minerals may be used for cultivating several other microbial groups for production of various important metabolites at commercial levels, viz., enzymes, polysaccharides, organic acids, and therapeutics.

## Conclusions

Jackfruit is an excellent source of a wide variety of nutrients, including carbohydrates, proteins, vitamins, minerals, dietary fiber, and phytochemicals. Studies that were carried out in the past revealed that jackfruit waste is also an excellent source for a wide variety of eco-friendly industrial products like biofuels, biomaterials, pharmaceuticals, food and feed additives, and a wide variety of other products. The process of converting jackfruit biomass into value added products involves the utilization of physico-chemical, biological and innovative green pretreatment methods. It is essential to investigate more genetically and biotechnologically advanced strategies in order to make the use of jackfruit wastes more functional and directed. Additionally, more research advancements are necessary to change the ways in which biorefineries currently produce a variety of value added products.

## Author contributions

NP: conceptualization and reviewing and editing. SS, RS, PS, PKS, SB, and BT: writing—original draft, reviewing, editing, and drawing figures. RG: reviewing and editing. MT: writing—original draft, reviewing, editing, and finalize the manuscript. All authors have read and agreed to the published version of the manuscript.

## Conflict of interest

The authors declare that the research was conducted in the absence of any commercial or financial relationships that could be construed as a potential conflict of interest.

## Publisher's note

All claims expressed in this article are solely those of the authors and do not necessarily represent those of their affiliated organizations, or those of the publisher, the editors and the reviewers. Any product that may be evaluated in this article, or claim that may be made by its manufacturer, is not guaranteed or endorsed by the publisher.
